# Bone Substitutes for Peri-Implant Defects of Postextraction Implants

**DOI:** 10.1155/2013/307136

**Published:** 2013-12-12

**Authors:** Pâmela Letícia Santos, Jéssica Lemos Gulinelli, Cristino da Silva Telles, Walter Betoni Júnior, Roberta Okamoto, Vivian Chiacchio Buchignani, Thallita Pereira Queiroz

**Affiliations:** ^1^Department of Oral Biology Postgraduation, Universidade do Sagrado Coração (USC), Bauru, SP, Brazil; ^2^School of Implantology of Cuiaba, Brazil; ^3^Department of Basic Sciences, School of Dentistry of Araçatuba, SP, Brazil; ^4^Department of Health Sciences, Implantology Post Graduation Course, Dental School, University Center of Araraquara, UNIARA, SP, Brazil

## Abstract

Placement of implants in fresh sockets is an alternative to try to reduce physiological resorption of alveolar ridge after tooth extraction. This surgery can be used to preserve the bone architecture and also accelerate the restorative procedure. However, the diastasis observed between bone and implant may influence osseointegration. So, autogenous bone graft and/or biomaterials have been used to fill this gap. Considering the importance of bone repair for treatment with implants placed immediately after tooth extraction, this study aimed to present a literature review about biomaterials surrounding immediate dental implants. The search included 56 articles published from 1969 to 2012. The results were based on data analysis and discussion. It was observed that implant fixation immediately after extraction is a reliable alternative to reduce the treatment length of prosthetic restoration. In general, the biomaterial should be used to increase bone/implant contact and enhance osseointegration.

## 1. Introduction

Although alveolar repair after tooth extraction can be conducted by blood clot, this repair is not complete due to physiological resorption [1]. Studies demonstrated that vertical and horizontal dimensions are reduced around 11–22% and 29–63%, respectively, due to alveolar resorption after 6 months following tooth extraction [2]. This atrophy is more intense in the buccal surface (about 0.8 mm) during the first 3 months [3].

The insertion of immediate implants in atrophic sockets is a challenge to achieve satisfactory esthetics and function [4]. In this sense, in 1976, Schulte and Heimke [5] presented the immediate implants that are placed in fresh sockets.

However, the diastasis observed between bone and implant after dental extraction may influence osseointegration [6]. So, autogenous bone grafts and/or biomaterials have been used in those gaps to correct bone defects and provide appropriate stability.

Considering the importance of stability of immediate implants, this study presented a literature review about the most common biomaterials used for immediate dental implants.

## 2. Material and Method

The inclusion criteria assumed the studies published in English from 1969 to 2012 searched at Medline (Pubmed) and Bireme databases. The keywords “dental implant,” “osseointegration,” “postextraction,” “bone substitute,” “fresh extraction sockets,” “immediate implant,” “bone repair,” “bone modeling,” “dehiscence,” “dimension,” and “ grafting” were used for searching.

**Figure 1:**
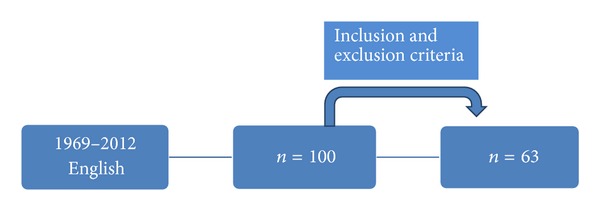
Method flowchart.

The search was based on scientific researches published in English including systematic reviews and also animal and human studies. The exclusion criteria were case reports and discussion articles. After analysis, 63 studies were selected according to the inclusion criteria. The results were based on data analysis and discussion. (Figure 1)

## 3. Literature Review

### 3.1. Gap Dimension

The distance between bone and implant is called peri-implant gap. The fresh socket is wider than the implant diameter, which causes the peri-implant gap that influences stability and osseointegration [6, 7].

In 1977, Schenk and Willenegger [8] conducted a study on rabbits and observed the lack of complete bone formation with peri-implant gaps wider than 1.0 mm. In 1988, Carlsson et al. [9] used the same experimental model to compare 3 values of peri-implant gap between bone and implant (group A—0 mm, group B—0.35 mm, and group C—0.85 mm) and observed residual gaps in groups B and C at 6 and 12 weeks after surgery.

In 1999, Akimoto et al. [10] placed postextraction implants in dogs and evaluated the repair of peri-implant gaps from 0.5 to 1.4 mm after 12 weeks. The results demonstrated that the defect size is inversely proportional to the bone/implant contact. However, Botticelli et al. [6] performed a similar study and found complete bone neoformation and osseointegration in defect with 1.0 mm after 16 weeks.

### 3.2. Autogenous Bone

Autogenous bone corresponds to bone graft obtained from the same individual. It is considered the gold standard for filling of bone defects since it allows (I) *osseointegration*: direct contact with bone tissue without fibrous tissue [11]; (II) *osteoconduction*: support to bone growth [11]; (III) *osteoinduction*: differentiation of mesenchymal cells of surrounding tissue (receptor site) into osteoblastic cells [12]; and (IV) *osteogenesis*: bone neoformation by osteoblastic cells present in the graft material [12]. Although few mature osteoblasts survive to grafting, precursor cells are responsible for the osteogenic potential [12].

Autogenous grafts are presented as blocks or particles and can be used isolated or associated with allogenic or alloplastic grafts. The donator area can be mentonian region, retromolar area, maxillary tuberosity, iliac crest, rib, cranium, tibia, and fibula [13].

Several studies evaluated peri-implant bone defects filled with autogenous bone. In 2013, Al-Sulaimani et al. [14] evaluated the success rate of immediate implants associated with autogenous bone for filling of the peri-implant gap. In this study, beagle dogs were used for insertion of implants immediately after extraction of right and left maxillary and mandibular lateral incisors. The gaps were filled with blood clot (control) and autogenous bone (experimental) and better results were found for the autogenous bone graft.

Nevertheless, this graft may cause morbidity in the donator area, hematoma, edema, infection, and vascular and nerve lesions. In addition, this technique spends more time for surgical procedure and it is limited for large reconstructions [15]. So, biomaterials have been suggested as an alternative to solve those limitations and reduce the gap between bone and implant.

### 3.3. Mineralized Bone Tissue

The matrix of mineralized bone tissue is composed by deproteinized bone tissue. It has been widely used for preservation of alveolar ridge dimension after tooth extraction, filling of bone defects near to natural teeth, and also during maxillary sinus lift [16–21].

In a study in monkeys, molars and premolar were extracted for fixation of titanium implants after 3 months. Peri-implant defects with 2.5 mm in width and 3.0 mm in height were filled with blood clot, polytetrafluorethylene membrane, Bio-oss, and Bio-oss with membrane. The histological analysis after 6 months revealed that Bio-Oss exhibits osteoconductive capacity and should be used for reconstruction of peri-implant bone defects [17].

Hockers et al. [18] conducted a similar study including one group with autogenous bone and observed that the bone grafts were integrated to the bone tissue.

Caneva et al. [19] used bone substitutes to fill the the gap between bone and implant. The effect of bone fillers (magnesium-enriched hydroxyapatite) on preservation of the alveolar bone around immediate implants was evaluated in a dog study. Implants with a sandblasted acid etched surface were placed into the fresh extraction sockets bilaterally into the dogs' jaws. Magnesium-enriched hydroxyapatite was placed at test sites,while the control sites did not receive augmentation materials. After 4 months of healing, the animals were sacrificed. Histomorphometric evaluations showed that the alveolar bony crest outline was maintained to a higher degree at the buccal bone wall of the test sites (loss: 0.7 mm) compared with the control sites (loss: 1.2 mm), even though this difference did not reach statistical significance.

In another experimental study Caneva et al. [20] explored the effect of GBR based on deproteinized bovine bone mineral on alveolar ridge preservation and the reparation of defects around osseointegrated implants. The authors concluded that the application of DBBM concomitant with a collagene membrane contributed in improving bone regeneration in the defects.

Barone et al. [21] showed that regenerative techniques (GBR) were able to limit resorption of the alveolar crest after implant placement in a fresh extraction socket, tooth extraction.

Hsu et al. [22] in an experimental study instead demonstrated that the placement of implants and deproteinized bovine bone mineral into fresh extraction sockets results in significant buccal bone loss and low osseointegration.

Other clinical studies [23–25] used GBR techniques to fill the gap between bone and implant.

### 3.4. Mineralized Bone Tissue with Addition of 10% Porcine Collagen

This material is composed by mineralized bovine bone matrix with addition of 10% porcine collagen (Bio-oss Collagen, Geistlich). This biomaterial is indicated for filling of extraction sockets, periodontal defects, and maxillary sinus lifting [26].

Araújo et al. [27] conducted an animal study with filling of extraction sockets with Bio-oss collagen. Biopsy and histometric analysis were performed after 3 months and demonstrated that the biomaterial promoted formation of bone tissue, maintained the dimension of alveolar walls, and preserved the alveolar crest profile.

In 2009, the same authors [28] performed a similar study for evaluation after 2 weeks. The results showed delayed alveolar repair with bone neoformation only at apical and lateral walls.

Wong and Rabie [29] conducted a study in rabbits to compare the amount of bone produced by Bio-Oss collagen and collagen matrix. Eighteen bone defects (5.0 mm × 10.0 mm) were created in the parietal bone of the rabbits and filled with Bio-Oss collagen, collagen matrix and blood clot. Biopsies were removed after 14 days for histological analysis. The authors concluded that the Bio-Oss collagen presented better results for bone neoformation in comparison to the collagen matrix while no bone was formed with the blood clot.

The study of Araújo et al. [30] is the only study evaluating peri-implant defects filled with Bio-oss collagen. In this paper, dogs were used to evaluate bone repair after fixation of immediate implants and insertion of mineralized bovine bone with addition of 10% porcine collagen. Biopsies were obtained after 6 months for histological analysis. The authors found that the presence of Bio-oss collagen changed the healing process of hard tissue, which improved bone/implant contact.

### 3.5. Beta-Tricalcium Phosphate

The *β*-tricalcium phosphate has been considered a material with excellent results since it is absorbable, osteoconductive, and nonosteoinductive [1]. Animal [26, 31–33] and human [34] studies demonstrated that this material supported bone neoformation.

In 2013, Daif [35] conducted a study to evaluate the influence of *β*-tricalcium phosphate on bone density surrounding immediate dental implants using helical computer tomography. Twenty-eight patients were selected and divided into two groups: (I) no filling and (II) filling with beta-TCP in the peri-implant defect. Tomography was obtained after 3 and 6 months and showed that the *β*-tricalcium phosphate increased bone density in the bone defect of immediate dental implants.

Recently, some industries developed a synthetic bone substitute composed by a homogeneous mixture of 60% of hydroxyapatite (HA) and 40% of beta-tricalcium phosphate [36]. Although the HA is resistant to physiological resorption [37], its osteoconductive capacity remains uncertain [38, 39]. On the other hand, the *β*-tricalcium phosphate is absorbed slowly and it is considered an osteoconductive material [40]. Thus, the HA maintains the gap while the *β*-tricalcium phosphate is absorbed to promote bone regeneration simultaneously [41].

In 2004, Boix et al. [42] evaluated the efficacy of this material in peri-implant defects in dogs and concluded that the biomaterial generated significant increase in bone regeneration surrounding the dental implant.

## 4. Discussion

**Figure 2:**
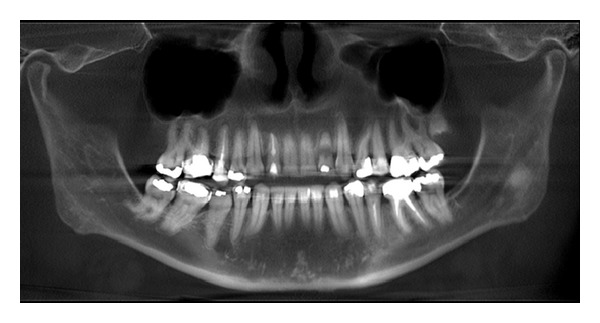
Initial panoramic radiograph.

**Figure 3:**
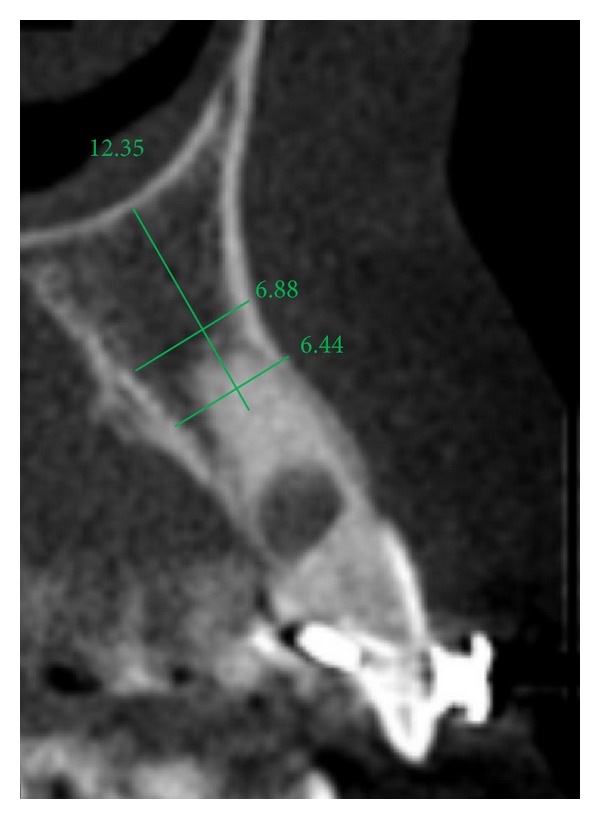
Initial computed tomography.

**Figure 4:**
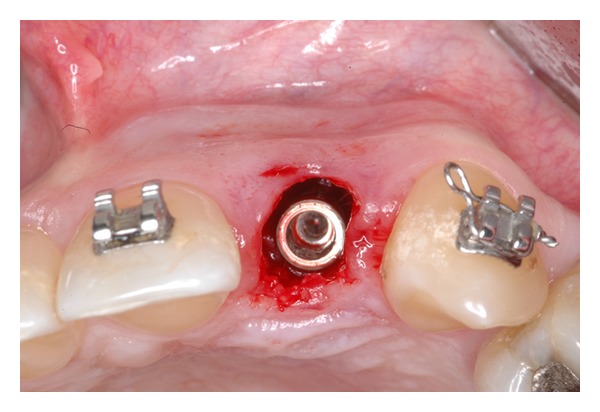
Peri-implant defect.

**Figure 5:**
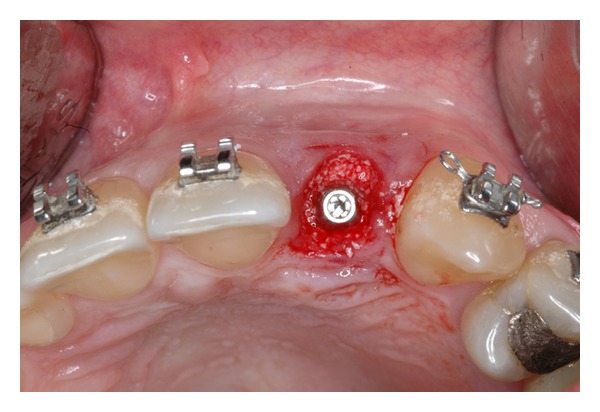
Peri-implant defect filled with biomaterial.

**Figure 6:**
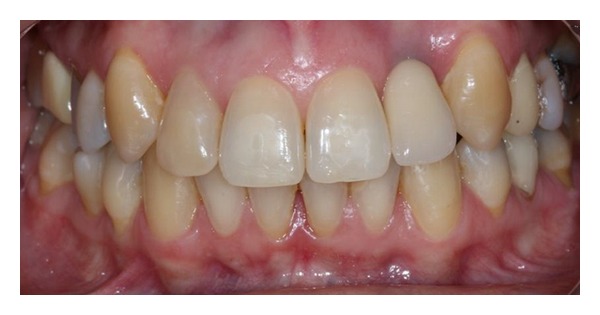
Esthetic restoration.

**Figure 7:**
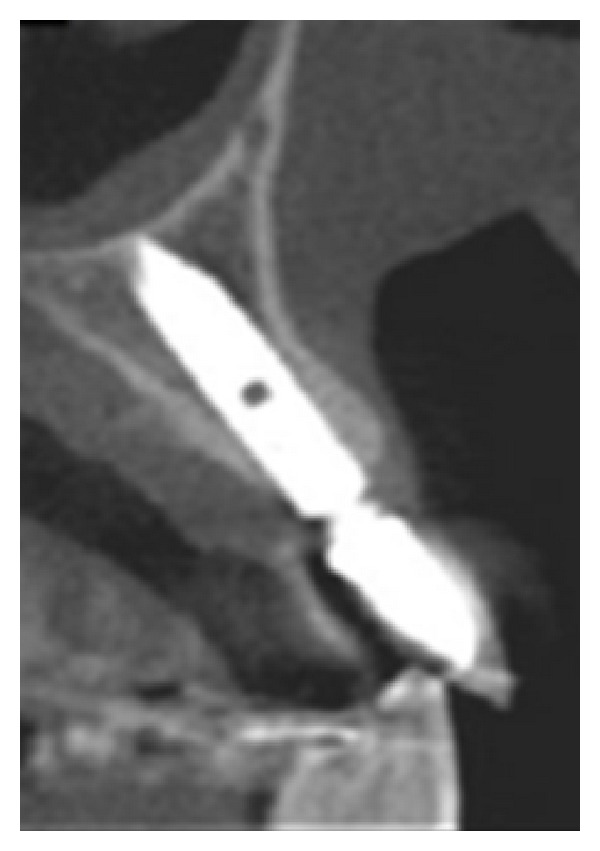
Final computed tomography.

The extraction socket is usually wider than the implant diameter, which results in a gap between the cervical region of the implant and the bone tissue (Figures 2, 3, and 4). Although this gap can be restored by maintenance of blood clot [43], the use of biomaterials is indicated to preserve alveolar ridge dimensions and promote repair [36] (Figure 5). In addition, the insertion of biomaterials simultaneously to the implant fixation improves functional and esthetic restoration of the stomatognathic system (Figures 6 and 7).

Several animal [6, 7, 30, 44, 45] and human [34, 39, 46–54] studies were conducted to evaluate the reconstruction of the peri-implant gap with biomaterials through clinical follow-up, histology, imaging, and immunohistochemistry. However, few of the literature reviews about biomaterials for peri-implant defects were found [55].

The ideal bone graft should present limited source, lack of morbidity in the donator site, no risk to disease transmission, efficient bone repair, immediate stability, versatility, easy manipulation, appropriate lifetime, and accessible cost [56].

The autogenous bone is considered the first option for bone reconstruction in implantology since it presents characteristics of the ideal graft. However, this approach requires longer surgical procedure and may not obtain enough bone volume [57]. So, alternative treatments have been suggest for peri-implant reconstruction.

Jensen et al. [33] compared the performance of autogenous bone, *β*-tricalcium phosphate, and anorganic bovine bone by histological and histomorphometric analyses in pigs. The authors observed greater efficacy for the autogenous bone in comparison to the other grafts.

On the other hand, Hockers et al. [18] compared grafting with autogenous bone and demineralized bovine bone for reconstruction of peri-implant defects in dogs and found similar integration for both materials. Similarly, Santis et al. [58] concluded that the autogenous bone and demineralized bovine bone provided a high level of bone regeneration and satisfactory bone/implant contact for osseointegration.

In 2009, Benić et al. [59] performed a human study to assess the success rate of peri-implant defects reconstruction with autogenous bone, demineralized bovine bone, and association of both materials. No difference was observed between the groups after a 5-year follow-up.

Han et al. [60] compared bone regeneration in peri-implant defects of dogs according to the following groups: (I) no filling, (II) autogenous bone, (III) Bio-Oss collagen, (IV) Bio-Oss, (V) no filling and collagen membrane, (VI) autogenous bone and collagen membrane, (VII) Bio-Oss collagen and collagen membrane, and (VIII) Bio-Oss and collagen membrane. The authors concluded that reconstruction of peri-implant defect with bone substitutes associated with membrane or not increases the percentage of bone/implant contact.

Guerra et al. [61] conducted a study on rabbits to compare grafting with bovine bone, bovine bone associated with platelet-rich plasma, bovine bone protected by membrane, and blood clot. A higher percentage of bone/implant contact with bovine bone protected by collagen membrane was observed.

Jensen et al. used immunohistochemistry in dogs to evaluate the performance of Bio-Oss collagen and Bone-Ceramic and found that both biomaterials are great osteoconductive materials for bone repair [33]. However, Antunes et al. [62] evaluated repair with blood clot, autogenous bone, Bio-Oss, and Bone-Ceramic in dogs and observed lower stability with Bio-Oss after 2 months.

Wang and Lang [63] evaluated the more recent studies in animal and human about this topic and they concluded that implants placed into the fresh extraction sockets do not prevent the resorption of the alveolar bone. In the research that was conducted bone regeneration with implant post-extractive implants would notice minor alveolar bone resorption. Moreover, other bone substitutes were tested: magnesium-enriched hydroxyapatite, human demineralized bone matrix, and deproteinized bovine bone mineral have been shown to be effective in ridge preservation. Applying the guided bone regeneration principle using bone substitutes together with a collagen membrane has shown clear effects on preserving alveolar ridge height as well as ridge width. Soft tissue grafts or primary closure did not show a beneficial effect on preserving the alveolar bone.

## 5. Conclusions

Considering this literature review, the fixation of implants immediately after tooth extraction is a reliable alternative to reduce the treatment length for patient's rehabilitation. In general, this treatment requires the use of a biomaterial to increase bone/implant contact and enhance osseointegration.
